# Interfacing Cultured Neurons to Microtransducers Arrays: A Review of the Neuro-Electronic Junction Models

**DOI:** 10.3389/fnins.2016.00282

**Published:** 2016-06-21

**Authors:** Paolo Massobrio, Giuseppe Massobrio, Sergio Martinoia

**Affiliations:** Neuroengineering and Bio-nanoTechnology Laboratory, Department of Informatics, Bioengineering, Robotics, System Engineering, University of GenovaGenova, Italy

**Keywords:** CNT, electrical equivalent circuit, extracellular signal, FET, MEA, neuron, neuro-electronic junction, sealing conditions

## Abstract

Microtransducer arrays, both metal microelectrodes and silicon-based devices, are widely used as neural interfaces to measure, extracellularly, the electrophysiological activity of excitable cells. Starting from the pioneering works at the beginning of the 70's, improvements in manufacture methods, materials, and geometrical shape have been made. Nowadays, these devices are routinely used in different experimental conditions (both *in vivo* and *in vitro*), and for several applications ranging from basic research in neuroscience to more biomedical oriented applications. However, the use of these micro-devices deeply depends on the nature of the interface (coupling) between the cell membrane and the sensitive active surface of the microtransducer. Thus, many efforts have been oriented to improve coupling conditions. Particularly, in the latest years, two innovations related to the use of carbon nanotubes as interface material and to the development of micro-structures which can be engulfed by the cell membrane have been proposed. In this work, we review what can be simulated by using simple circuital models and what happens at the interface between the sensitive active surface of the microtransducer and the neuronal membrane of *in vitro* neurons. We finally focus our attention on these two novel technological solutions capable to improve the coupling between neuron and micro-nano transducer.

## Introduction

Signal recording systems (microtransducers) based on Multi-Electrodes Arrays (MEAs) and Field Effect Transistors (FETs) have been demonstrated as powerful tools for recording the electrical activity of networks of neurons cultured in *vitro* (Vassanelli and Fromherz, 1998; Taketani and Baudry, 2006). Under this experimental condition, neurons are directly coupled to the microtransducer by a neuro-electronic junction, and the neuronal electrical activity is then extracellularly recorded.

**Figure 1:**
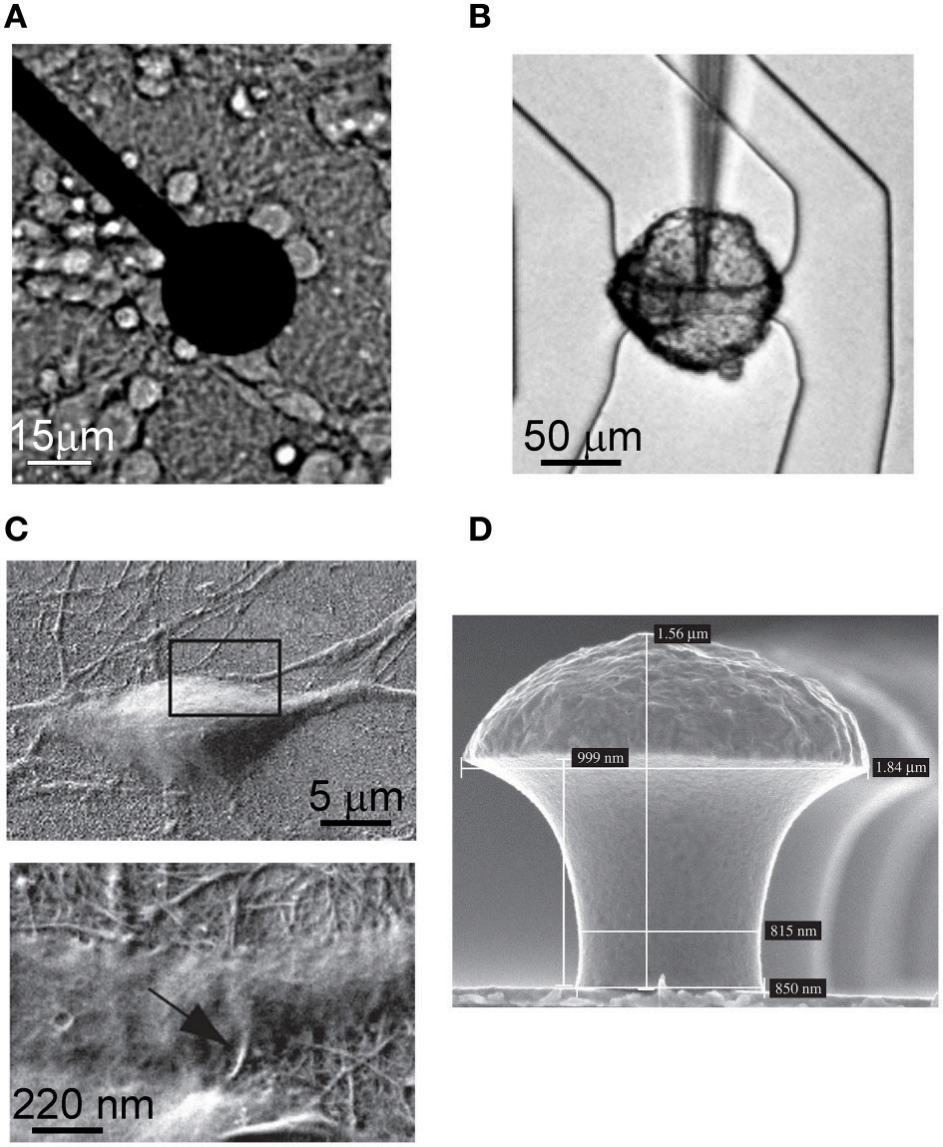
**Images of microtransducers coupled to different neuronal cultures. (A)** Metal microelectrode of a MEA covered with cortical neurons. **(B)** Cell body of a leech neuron coupled to a FET. (*Adapted from* Fromherz, 2003). **(C)** Hippocampal neuron grown on CNTs. The two magnifications show the intimate contact between the neuron and the CNTs. (*Adapted from* Mazzatenta et al., 2007). **(D)** Gold-spine microelectrode (*Adapted from* Hai et al., 2009).

The history of the microtransducer arrays as extracellular recording devices begins at the end of the 60's, when the first metal microelectrodes were adopted (Robinson, 1968). Thomas et al. (1972) introduced the first MEA in 1972. It consisted of platinized gold microelectrodes embedded onto a glass substrate and passivated by photoresist. This device allowed to record field potentials from spontaneous contracting sheets of cultured chick cardiomyocytes, but it was not able to record activity from a single cell. Only in the 80's, Pine and Gross (Pine, 1980; Gross et al., 1982) designed arrays made up of 32 electrodes able to record the electrophysiological activity of excitable cells, and validated this approach on neuronal networks. MEAs enable long-term neuron signal recording thanks to their non-invasive properties and, at the same time, allow applying external stimuli using the same recording electrodes. Figure 1A shows an optical image of a neuronal culture coupled to a single microelectrode.

A considerable contribution in the microtransducers field for electrophysiological neuronal activity recording was made by Fromherz's lab (Fromherz et al., 1991; Vassanelli and Fromherz, 1998). He pointed out that insulated gate FETs are also able to detect the transient extracellular voltage beneath a single neuron attached, with its cell membrane, to the gate insulator of the FET. The neuron activity leads to ionic and displacement currents flowing through the attached membrane, resulting into an extracellular voltage drop along the narrow cleft between the membrane and the gate insulator. The change of the extracellular voltage induced by the neuron gives rise to an electric field across the insulator which modulates the drain-to-source current of the FET; this current, translated into a voltage, describes the extracellular recorded signal probed by the microtransducer. Figure 1B depicts the cell body of a neuron of leech coupled to a FET.

The latest contributions in the microtransducers field for electrophysiological applications were devoted to increase the coupling with the neuronal membrane. Starting from the beginning of 2000s, some studies showed carbon-nanotubes (CNTs; Iijima, 1991) can provide a good surface for neuronal cell adhesion and growth, both on uniformly covered surfaces (Mattson et al., 2000) and on isolated CNTs (Gabay et al., 2005; Lovat et al., 2005). In Figure 1C, two different details of the intimate contact of hippocampal neurons grown on CNTs are shown: the excellent biocompatibility of the material and the tiny dimensions of the CNTs facilitate the coupling to the biological membranes. Recently, a very interesting contribution to enhance the quality of the recorded signal has come from Spira's lab. Figure 1D shows the proposed gold mushroom-shaped electrode (Hai et al., 2009) which allows to increase the coupling with the neuronal membrane, and to achieve an extracellular signal shape resembling the neuron action potential.

Independently of the type of microtransducer, its performance heavily depends on the nature of the interface (i.e., neuro-electronic junction) between its active sensitive surface and the cell membrane grown on it. Thus, modeling this interface is an important issue for researchers to efficiently simulate the cell-microelectrode system. The aim of this review is to present a characterization, by means of an equivalent electrical circuit approach, of the neuro-electronic junction in the experimental condition of *in vitro* neurons coupled to micro-/nano-transducers. This work is organized in two main sections: (a) a description of the biophysical phenomena at the basis of the neuro-electronic interface; (b) a description of the most attractive developed electrical models of the “neuron-interface-microelectrode system” to simulate and understand the recorded extracellular neuronal signals.

The basic elements of the presented neuro-electronic junction model start from the Gouy-Chapman-Stern theory devised to describe the electrochemical reactions and ionic charge re-distributions at the solid-electrolyte interface (Bockris and Reddy, 1977; Bard and Faulkner, 1980). It is worth noticing this review presents the models of the neuron-interface-microelectrode system operating in the recording mode (i.e., microtransducers used only to record extracellular signals), and neglects the delivering mode operation (microtransducers used as electrical stimulation).

## Equivalent circuit of the neuron-microtransducer interface

**Figure 2:**
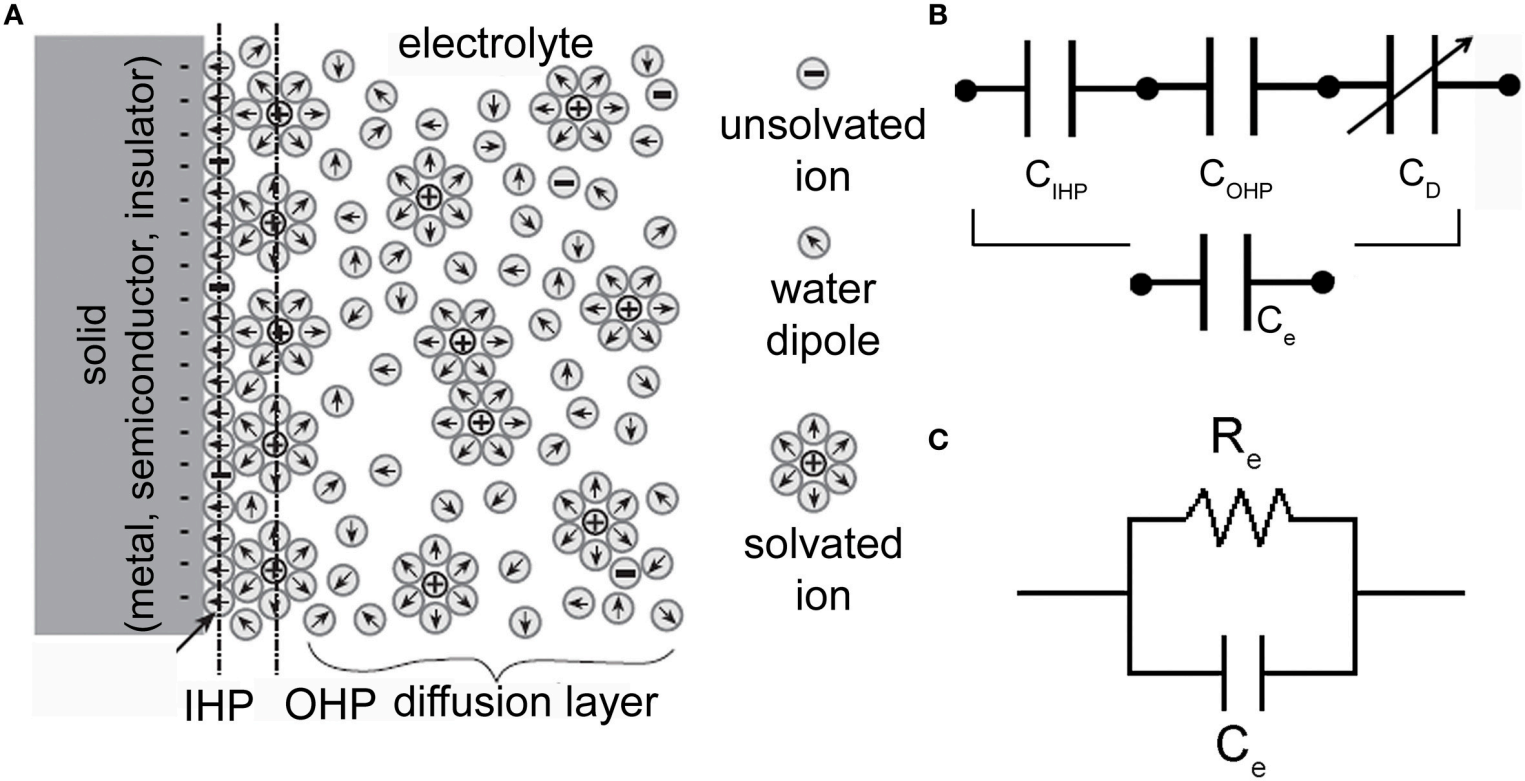
**Electrode-electrolyte interface and equivalent electrical circuits. (A)** Schematic representation of an electrode-electrolyte interface. (*Adapted from* Bockris and Reddy, 1977). **(B)** Series of three capacitors (corresponding to the IHP, OHP, and diffuse-layer) which models the charge distribution at the interface. **(C)** Electrical circuit of an electrode-electrolyte interface. *R*_*e*_ is the charge-transfer resistor and *C*_*e*_ is the double-layer capacitor.

When a solid (metal, semiconductor, insulator), and in particular an electronic conductor, i.e., an electrode, is placed into an ionic conductor, i.e., an electrolyte (a solution where charge is carried by the movement of ions), an electrified interface develops (Siu and Cobbold, 1979). Water dipoles orient themselves in the field within a layer at the electrode surface, forming what is known as the hydration sheath (a highly oriented layer of water molecules on the surface of the electrode). Solvated ions exist in a second layer outside this hydration sheath. If they do not penetrate the sheath, they form a plane of charge parallel to the electrode surface known as the Outer Helmholtz Plane (OHP). Their distribution is like a cloud of ions with a higher density near the electrode and a decaying away farther from the electrode surface. This ionic-cloud is referred to as the Gouy-Chapman diffuse-charge layer (GCL). Some ions are able to penetrate the hydration sheath and adhere to the electrode. The location of the plane from the electrode surface is considered to be the locus of absorbed-ion centers and is referred to as the Inner Helmholtz Plane (IHP). The properties of the space-charge distribution shown in Figure 2A may be summarized by an equivalent circuit representation (Figure 2B) made up of a series of three capacitors, each describing the charge distribution in the pertinent layer (i.e., IHP, OHP, and diffusion-layer). In addition, a “charge transfer” resistor *R*_*e*_ connected in parallel to the series of the three capacitors of Figure 2B (that from now named *C*_*e*_) has to be considered in the model. The final configuration is depicted in Figure 2C.

**Figure 3:**
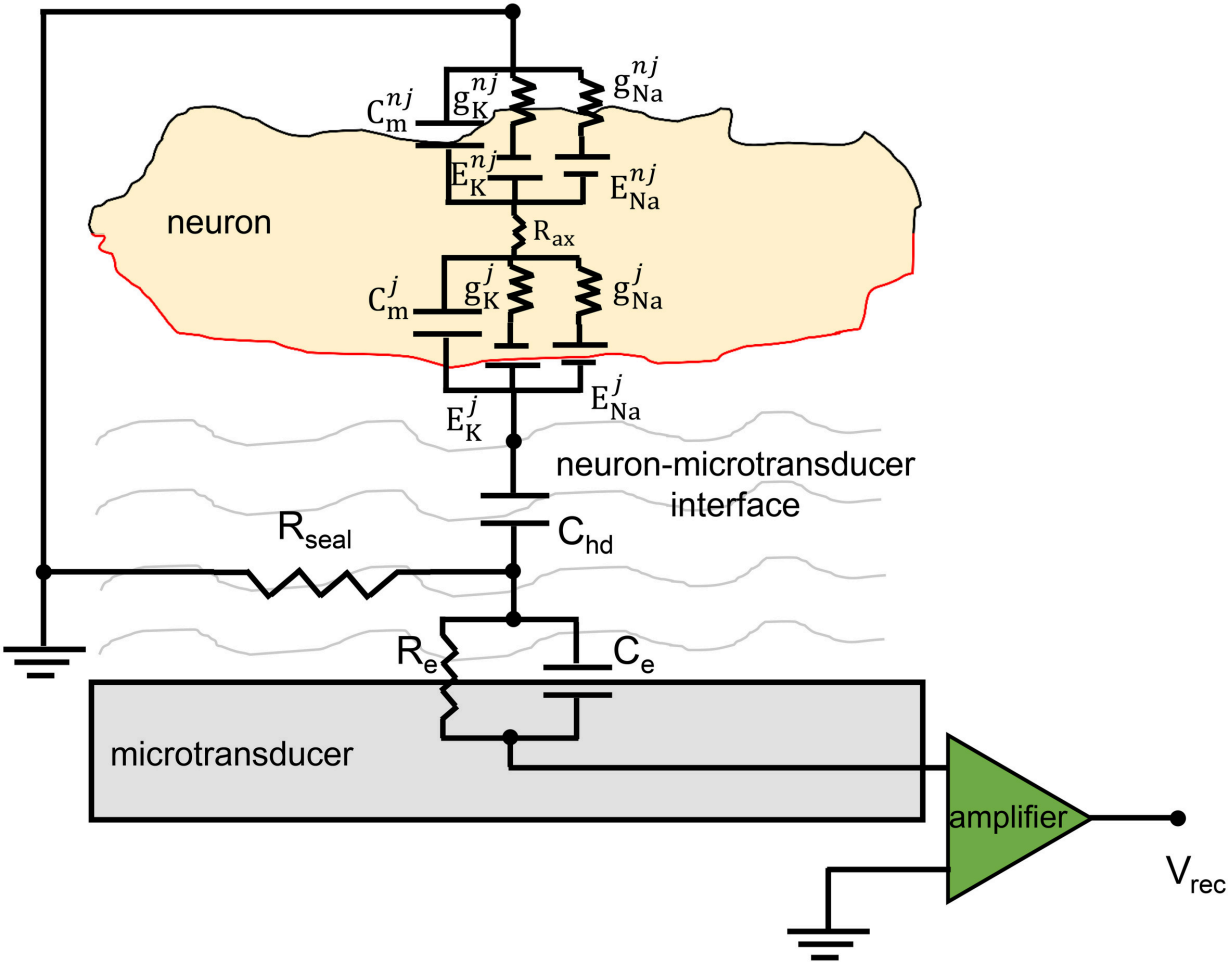
**Equivalent circuit of the neuro-electronic junction**. The schematics display the coupling between a neuron and a generic microtransducer. The neuro-electronic junction is described by the parallel *R*_*e*_–*C*_*e*_, the sealing (*R*_*seal*_) resistance and the capacitance *C*_*hd*_ (cf., the text for details). The electrophysiological intracellular activity is generated by the two parallels which model the sodium and potassium channels (*E*_*Na*∕*K*_ and *g*_*Na*∕*k*_ represent the Nernst potentials and the conductances); *C*_*m*_ describes the capacitive behavior of the neuronal membrane. The membrane capacitance is split in two parts: the “junctional” one, (red) whose electrical components are individuated with the superscript *j*, and the “non-junctional” one (black, superscript *nj*).

The equivalent circuit of the neuron-to-microtransducer junction is shown in Figure 3. This figure highlights the presence, in the cleft between the neuron and the microtransducer (passive microelectrode or FET-based device), of a circuit which couples the biological membrane of the electrogenic cell to the recording stage, making the signal recording possible. As Figure 3 points out, the coupling circuit model is very simple and made up of passive electrical components (i.e., resistors and capacitors).

The physical meaning of the model components is as follows. The *sealing resistance* (*R*_*seal*_) models how much the cell is attached to the microtransducer, that is, it describes the separation of the neuron and the recording device sensitive area which results into an extended cleft of electrolyte; it is in parallel to the microelectrode surface (cellular membrane). The simplest formulation for evaluating this resistance is:

(1) $$R_{seal}=\frac{\rho_{s}}{d}\;\cdot \;\delta$$

where ρ_*s*_ is the resistivity of the electrolyte solution (for normal saline ρ_*s*_ = 0.7 Ωm), *d* is the average neuron-to-microtransducer distance [that can be experimentally evaluated by means of fluorescence interferometry (Braun and Fromherz, 1998)], δ is a surface overlapping coefficient that takes into account the percentage of the microtransducer sensitive area covered by the neuron. Its expression can be evaluated depending on the different layouts involving the neuron and the microtransducer areas. In particular, we can write:

(2) $$\begin{array}{l} \delta =\;\frac{A_{neuron}}{A_{microtransd}}\;for\;A_{neuron}\;<\;A_{microtransd}\\ \delta =\;1\;for\;A_{neuron}\;\geq \;A_{microtransd} \end{array}$$

In Equation (2), *A*_*microtrasd*_ and *A*_*neuron*_ represent the microtransducer and the neuronal membrane areas, respectively.

Modeling the sealing resistance, a key-parameter in explaining the recorded signals, fundamentally involves the microtransducer surface covered by the cell. A wide and detailed characterization of such a component was performed by Braun and co-workers (Braun and Fromherz, 2004): they estimate the value of *R*_*seal*_ by applying sinusoidal voltage stimulation to the insulator of a FET, and by imaging the voltage change across the attached cell membrane with fluorescent voltage-sensitive dye (VSD). The phase map of voltage change was fitted with a planar core-coat conductor model, using the seal resistance as a free parameter. They found, for rat neurons, a value of 14 MΩ which is consistent with evaluations obtained from Equation (1).

The spreading resistance (*R*_*spread*_) models the signal loss due to the distance between the neuron and the microelectrode; it is placed perpendicularly to the microelectrode surface (cellular membrane). For a circular microelectrode, the value of *R*_*spread*_ can be calculated, according to (Newman, 1966):

(3a) $$R_{spread}=\frac{\rho_{s}\;\cdot \;\sqrt{\pi }}{4\;\cdot \;\sqrt{A_{microtransd}}}$$

On the other hand, when the rectangular shape of the microtransducer is considered, the value of *R*_*spread*_ can be calculated, according to (Kovacs, 1994):

(3b) $$R_{spread}=\frac{\rho_{s}\;\cdot \;\ln\left(4\;\cdot \;\frac{W_{microtransd}}{L_{microtransd}}\right)}{\pi \;\cdot \;W_{imicrotransd}}$$

where *W*_*microtransd*_ and *L*_*microtransd*_ are the width and length of the sensitive area of the microtransducer, respectively. However, with reference to the results obtained from Martinoia et al. (2004) and Massobrio et al. (2007) which showed no significant modifications of the extracellular signal shape by varying, also within a wide range the values of *R*_*spread*_, this component is not introduced in the circuit model of the neuro-electronic junction.

*C*_*hd*_ (neuron membrane-to-electrolyte capacitance) models the polarization layers of the electrolyte solution in front of the neuron membrane and the capacitive part associated with the protein-glycocalyx complex attached to the portion of the cell membrane in contact with the microelectrode. From the double-layer theory (Yates et al., 1974) and the GCL model, the capacitance *C*_*hd*_ is defined as the series of the Helmholtz layer capacitance:

(4) $$C_{Helm}=\frac{\left(\varepsilon_{IHP}\cdot \;\varepsilon_{0})\;\cdot \;(\varepsilon_{OHP}\cdot \;\varepsilon_{0}\right)}{(\varepsilon_{OHP}\cdot \;\varepsilon_{0})\;\cdot \;d_{IHP}+(\varepsilon_{IHP}\cdot \;\varepsilon_{0})\;\cdot \;d_{OHP}}\cdot \;A_{cont}$$

and the Gouy-Chapman or diffuse-layer capacitance:

(5) $$C_{Gouy}=\frac{q\;\cdot \;\sqrt{2\;\cdot \;\varepsilon_{r}\cdot \;\varepsilon_{0}\cdot \;k\;\cdot \;T\;\cdot \;C_{b}}}{k\;\cdot \;T}\;\cdot \;A_{cont}$$

Equation (5) holds for the potential across the diffuse-layer much < 2 times the thermal voltage (*kT*/*q*).

In Equations (4, 5), ε_*IHP*_ and ε_*OHP*_ are the inner and OHP relative dielectric constants, respectively; *d*_*IHP*_ is the neuron to non-hydrated ion distance; *d*_*OHP*_ is the neuron to hydrated ion distance; ε_0_ is the dielectric permittivity of free space; ε_*r*_ is the diffuse-layer relative dielectric constant; *A*_*cont*_ = (*A*_*microtransd*_. δ) is the contact area neuron-microtransducer surface; *k* is the Boltzmann's constant; *T* is the absolute temperature; *q* is the electron charge; *C*_*b*_ is the bulk concentration.

Finally, the electrolyte-to-microtransducer interface is modeled by a resistive-capacitive parallel circuit derived from the physico-chemical considerations depicted in Figure 2. Referring to Figure 3, such interface is modeled by the capacitor *C*_*e*_ which takes into account the capacitance of the electric double-layer, and the resistor *R*_*e*_ (leakage resistor) which describes the flow of the charge carriers crossing the electric double-layer.

**Figure 4:**
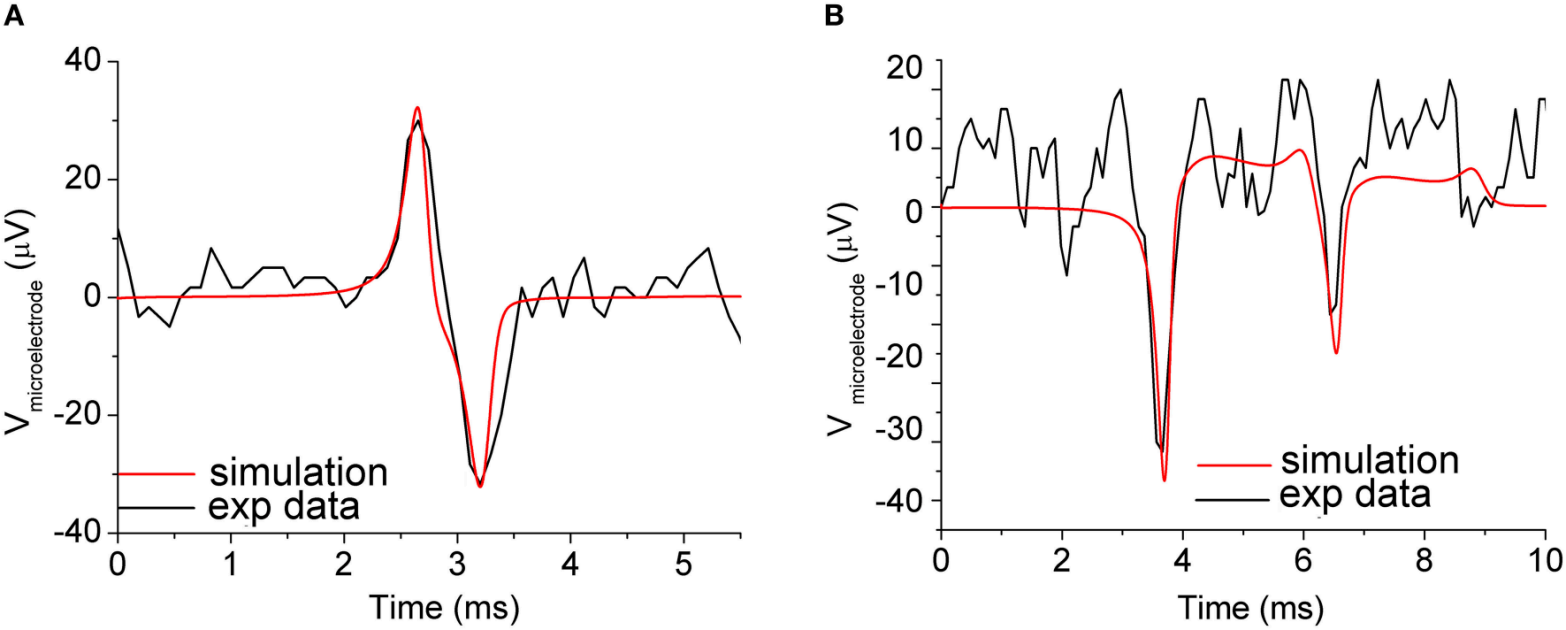
**Simulation of the neuron-to-microelectrode junction. (A)** Neuron-to-microelectrode coupling in a one-to-one correspondence: experimental measurements (black line), and simulations results (red line). Coupling parameters set: *R*_*seal*_ = 5 MΩ and *C*_*hd*_ = 17.45 pF. **(B)** Two synaptically connected neurons coupled to a microelectrode: experimental measurements (black line), and simulations results (red line). Coupling parameters set: *R*_*seal*__1_ = 30 MΩ, *R*_*seal*__2_ = 10 MΩ, *C*_*hd*__1_ = 17.45 pF, *C*_*hd*__2_ = 10 pF, which implies neuron-to-microelectrode compartments distances *d*_1_ = 12 nm and *d*_2_ = 35 nm, respectively. The subscripts 1 and 2 refer to the first and second neuron coupled to the compartmentalized microelectrode. *Adapted from* Martinoia et al. (2004).

To test the reliability of the described neuron-to-microelectrode junction model, the electric activity of cortical neurons from rat embryo, experimentally recorded by a 30 μm diameter gold metal microelectrode, was simulated. The neuron interfacing the microelectrode was represented by a specific compartment with an average area of about 350 μm^2^, resulting in a surface overlapping coefficient δ = 0.5. The value of *C*_*hd*_ for the neuron-to-microelectrode junction model were calculated according to Equations (4, 5), assuming: ε_*IHP*_ = 6, ε_*OHP*_ = 32, *d*_*IHP*_ = 0.3 nm, *d*_*OHP*_ = 0.7 nm, ρ_*s*_ = 0.7 Ωm, *C*_*b*_ = 150 mM, *A*_*cont*_ = 353.5 μm^2^, thus obtaining *C*_*hd*_ = 17.45 pF; the value of *R*_*seal*_ was used as a fitting parameter. To simulate the experimental recorded signals shown in Figure 4A (black line), *R*_*seal*_ was set to 5 MΩ, corresponding to an average neuron-to-microelectrode distance *d* = 70 nm, in good agreement with the experimental results found in Vassanelli and Fromherz (1999). The simulation results shown in Figure 4A concern the neuron-to-microelectrode coupling in a one-to-one correspondence, condition that can be experimentally obtained by means of micro-patterning technique (Shein et al., 2009). However, in the most common experimental conditions (i.e., dense homogeneous neuronal networks), a few neurons (soma and-or neurites) are coupled to a single microelectrode and the recorded signal is a combination of signals coming from different neurons (Maeda et al., 1995). This makes the recorded extracellular shape more complex (Figure 4B, black line), and only using spike-sorting techniques it could be possible to individuate the sources (Pedreira et al., 2012).

The simulated signal of Figure 4B (red line) was accomplished by considering a compartmentalized microelectrode to follow the topography of the network and to take into account different coupling conditions. By tuning *R*_*seal*_ and *C*_*hd*_ within reasonable range of values, we could achieve a good overlap between experimental and simulated signal. It should be noted that, in this case, the estimated couplings distances are 12 and 35 nm (among different portions of the microelectrode and the adhering neurons) and even if those values justify the different amplitude of the signal, they should be considered as hypothetical limiting values. From these simulations, and from other experimental works (e.g., Braun and Fromherz, 1998; Sorribas et al., 2001), it emerges how the neuron-microtransducer coupling is mediated by the extracellular clefts. By means of fluorescence interferometry experiments, Braun and Fromherz measured a distance ranging from 60 to 105 nm (Braun and Fromherz, 1998) by using laminin as adhesion factor. These distances, although rather wide, allow to effectively record supra-threshold signals with reasonable coupling factors. More recently, Thakore and coworkers measured more narrow clefts between the cell membrane and the microtransducer surface, finding a possible lower bound of about 20 nm (Thakore et al., 2012). Although the Debye length is smaller than the neuron-microelectrode cleft, the ionic distributions of the electrode-electrolyte interface (cf., Section Equivalent Circuit of the Neuron-Microtransducer Interface) extend across the entire interface, thanks to the presence of fixed charges associated with the glycocalyx matrix.

However, most of the attempts of the latest years are turned to increase such a coupling, minimizing the distance between cell membrane and microtransducer by using more complex structures as CNTs or engulfed mushroom-shaped microelectrodes as discussed in Section Increasing the Coupling.

The portions of the microelectrode stage, modeled by means of the microelectrode double-layer *R*_*e*_–*C*_*e*_ parallel, were connected together by the resistor *R*_*met*_ which models the low resistance of the metallic layer. The neuron-to-microelectrode coupling conditions were described by means of the seal resistors *R*_*seal*_, and of the capacitor *C*_*hd*_. More details can be found in Martinoia et al. (2004).

## Effects of the neuro-electronic junction coupling parameters on the recorded signal

**Figure 5:**
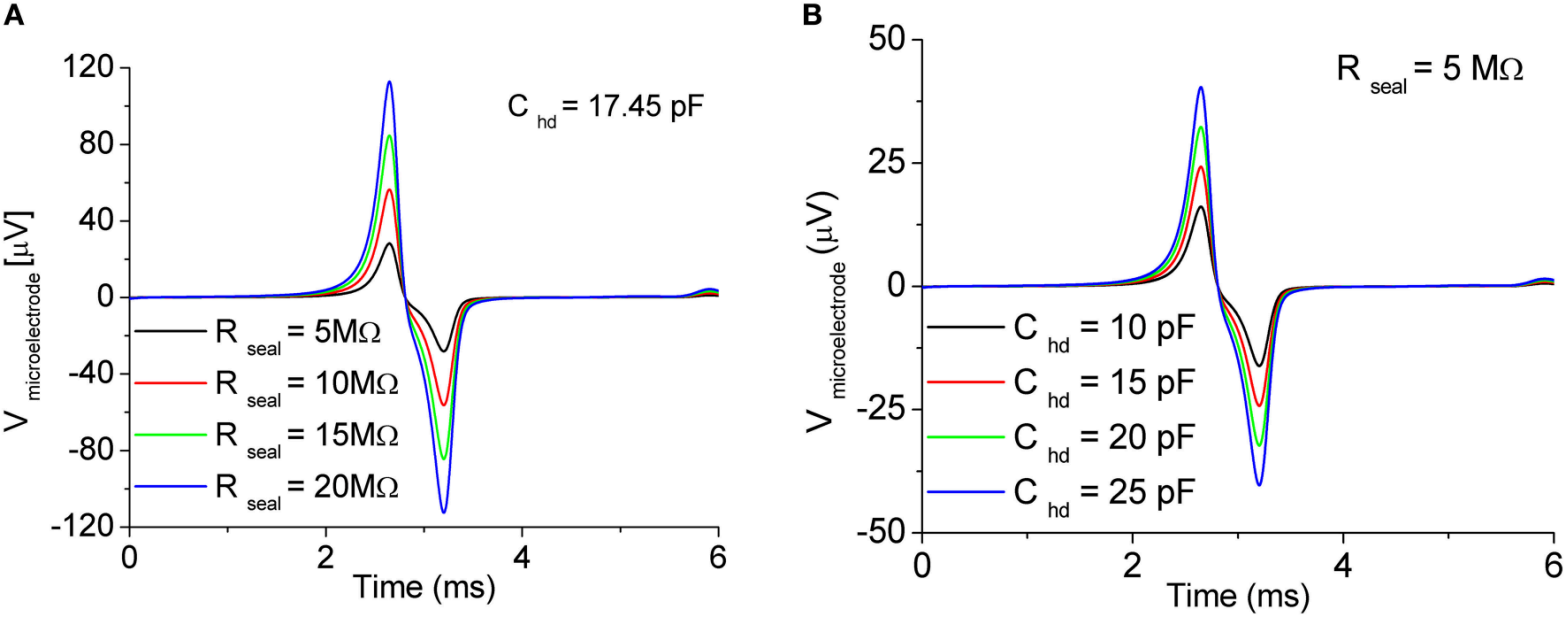
**Effect of the coupling parameters on the recorded signal**. Sweep of **(A)** sealing resistance *R*_*seal*_ and **(B)** Gouy-Chapman capacitance *C*_*hd*_. In both the cases, the effect is a reduction/amplification of the extracellular signal amplitude. *Adapted from* Martinoia and Massobrio (2004).

As examples of the influence of the coupling parameters on the signal shape, Figures 5A,B show the results of the simulations of the extracellular potential of one neuron coupled to one microelectrode at four values of the seal resistance *R*_*seal*_ (5, 10, 15, 20 MΩ) and at four values of *C*_*hd*_ (10, 15, 20, 25 pF), respectively. In Figure 5A, the value of *C*_*hd*_ is set at 17.45 pF, while in Figure 5B, *R*_*seal*_ is set at 5 MΩ.

By varying the values of *R*_*seal*_ and *C*_*hd*_, only slight changes in the shape of the recorded signals were obtained. On the other hand, being *R*_*seal*_ and *C*_*hd*_ dependent on the geometry (i.e., in particular on the distance) of the coupling between the neuron compartment and the recording microelectrode, the main influence of these two parameters affected the peak-to-peak signal amplitude. Possible changes in the shape of the extracellular signal (cf. Figure 4A, biphasic shape vs. Figure 4B, monophasic shape) are due to other factors such as possible migration of the trans-membrane channels directly coupled to the active area of the microtransducer (cf., Section Ion-Channels Migration at the Interface Promotes Different Signal Shapes).

## Ion-channels migration at the interface promotes different signal shapes

Experimental evidences show the recorded extracellular signals can display different shapes: in other words, although the intracellular action potential always presents the same stereotyped shape, the recorded signals may display a biphasic (Figure 4A) or a monophasic (Figure 4B) shape, and the negative/positive peaks position may be shifted in time. Fromherz and colleagues studied this particular behavior speculating that the changes in the signal shape could be due to a different density of the voltage-dependent channels (i.e., sodium and potassium) at the interface (Schatzthauer and Fromherz, 1998). By performing experiments where leech neurons (extracted from *Hirudo medicinalis*) were coupled to the active area of a FET, they found that the negative transient of the extracellular signal corresponds to an inward flow of sodium ions, while the positive response to an outward flow of potassium ions. The same authors simulated these conditions by using a circuit model equivalent to the one depicted in Figure 3.

**Figure 6:**
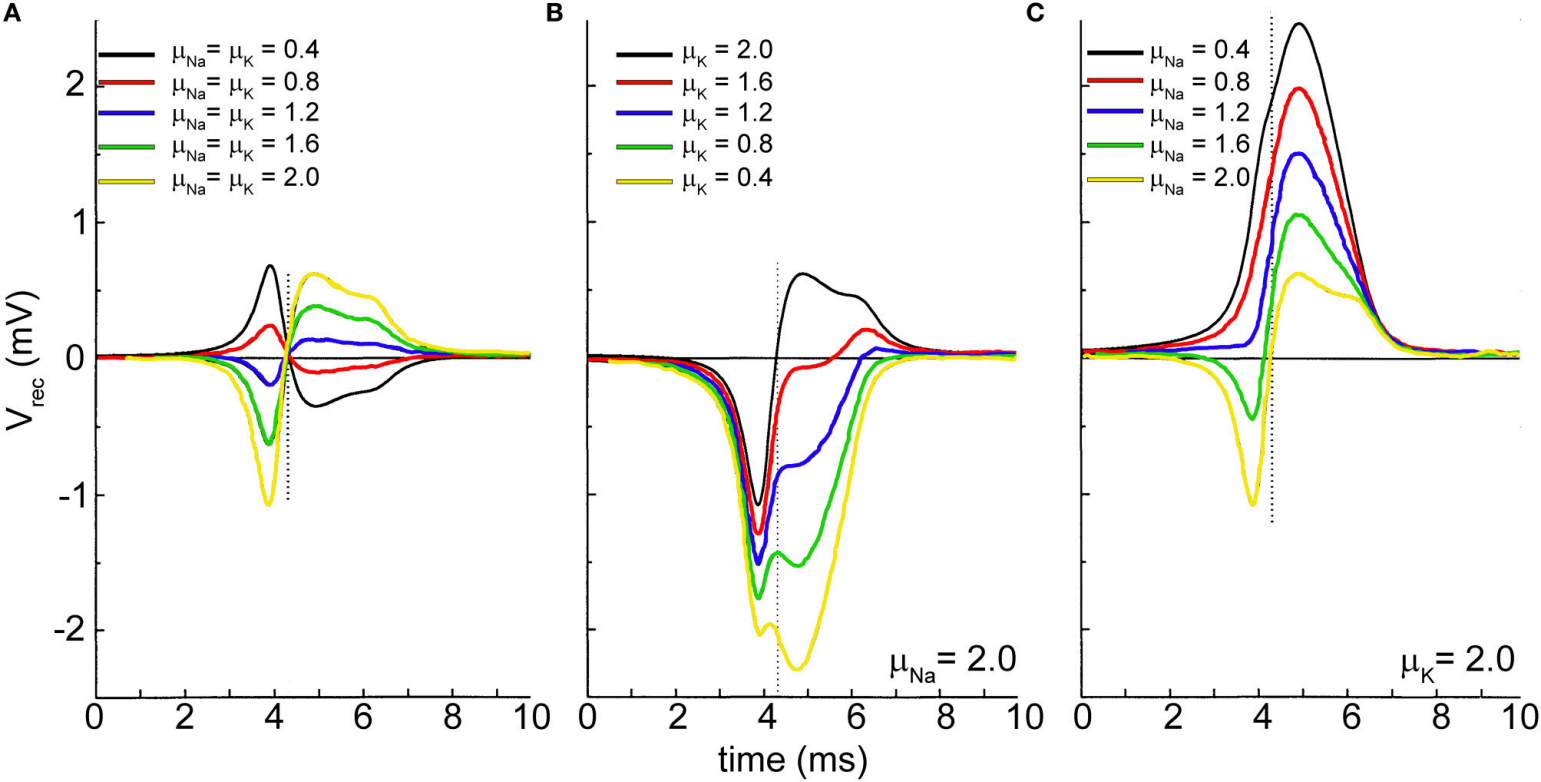
**Extracellular recorded signal shape affected by the sodium and potassium channels density at the neuron-microtransducer interface**. The maximum values of the sodium and potassium conductances are modulated by means of the factors μ_Na_ and μ_K_. **(A)** μ_Na_ = μ_K;_ **(B)** μ_Na_ kept constant with high value (μ_Na_=2.0); **(C)** μ_K_ kept constant with high value (μ_K_ = 2.0). *Adapted from* Schatzthauer and Fromherz (1998).

Figure 6 shows the effects of the simulations when the maximum values of the conductances (*g*_*Na*_ and *g*_*K*_) of the voltage-dependent sodium and potassium channels coupled to the microtransducer are increased/decreased by means of multiplicative factors (μ_*Na*_ and μ_*K*_). Figure 6A accounts for the condition when *g*_*Na*_ and *g*_*K*_ are increased/decreased by the same factors (μ_*Na*_ = μ_*K*_). If the channels density at the interface is higher than that in the uncoupled membrane (red, green, blue, and yellow lines of Figure 6A), the extracellular signal takes on a biphasic shape. In addition, the rising phase of the action potential corresponds to a negative voltage transient of the extracellular signal, while the positive phase corresponds to the membrane polarization. When *g*_*Na*_ and *g*_*K*_ are decreased (black and red lines of Figure 6A), the signal is overturned resembling a monophasic shape. In a second set of simulations, Fromherz and colleagues kept high the values of the sodium (Figure 6B) and potassium (Figure 6C) conductances, and made the other channel conductances sweep. From Figure 6B, one can observe the extracellular signal shape becomes monophasic when the potassium conductance is lower than the sodium one (μ_*K*_ ≤ 1.6); moreover, a further decreasing of the value of this conductance induces a negative transient in the falling phase of the action potential, until two negative peaks emerge (Figure 6B, green and yellow lines). Finally, keeping high *g*_*K*_, and sweeping *g*_*Na*_, the negative transient disappears, while a positive peak in the falling phase of the action potential is noticed, as high as the level of sodium conductance is lowered.

Considering that the channels expression in the neuronal membrane is a dynamic process, any kind of disturbance can influence the local distribution. From a bio-electrochemical point of view, phenomena such as diffusion and electrophoresis drive a channel migration; in addition, the possible interactions originated between cell membrane and adhesion factors contribute to change the physiological channels distribution (Angelides et al., 1988). From a computational point of view, a wide investigation of the relationship between channels distribution and extracellular recordings has been performed in 2002 by Buitenweg et al. (2002) who quantified and estimated, from the extracellular shape, the effect of the altered ionic channels distributions. However, the modeling strategy followed by the authors does not fall within the electrical-circuit based approach being based on finite-elements modeling.

## Increasing the coupling

In the latest years, attempts have been made to increase the quality of the recorded signals obtained from extracellular microtransducers. The use of these micro-devices deeply depends on the nature of the interface (coupling) between the cell and the sensitive active surface of the microtransducer. In the previous sections, it has been emphasized how the behavior of this interface depends on specific coupling parameters (i.e., sealing and spreading resistances, and double-layer capacitances), and how efforts have been focused to improve these coupling parameters and conditions. However, two of the major limitations in using these devices are the difficulty to record low-threshold signals (e.g., synaptic potentials) and the high dependence of the output signal on the microtransducer input impedance. As an example, 30 μm in diameter planar gold microelectrode exhibits an impedance of about 50 kΩ at 1 kHz. A reduction of the microelectrode dimensions entails an increase of the impedance (e.g., 10 μm in diameter planar gold microelectrode exhibits an impedance of about 400 kΩ at 1 kHz; data provided by Multi Channel Systems datasheet, www.multichannelsystems.com). On the other hand, a reduction of the dimensions of the sensitive area of the microtransducer is a strong requirement to record the activity of a single cell (one-to-one coupling) or for reaching a sub-cellular resolution. In the latest years, attempts have been performed for matching the requirement of the microtransducer dimension reduction (positive effect) and the resulting increase of the impedance, as well as the signal-to-noise-ratio (S/N) reduction (negative effects). Indeed, a possibility widely implemented since the 90's is related to the use of active devices (i.e., transistor-based structures) that allow greatly reducing the dimensions of the microtransducer (down to few micrometers) even if S/N is partly degraded (Fromherz, 2003; Hafizovic et al., 2007). Starting from 2000's, two main routes have been followed: (i) coating of carbon nanotubes (CNTs) on the active surface of the device; (ii) development of engulfed protruding nano-electrodes which guarantee a kind of “giga-sealing” with the neuronal membrane. In the next two sections, these approaches will be presented and discussed from the theoretical point of view.

### Carbon-nanotube coating improves both signal amplitude and firing frequency

**Figure 7:**
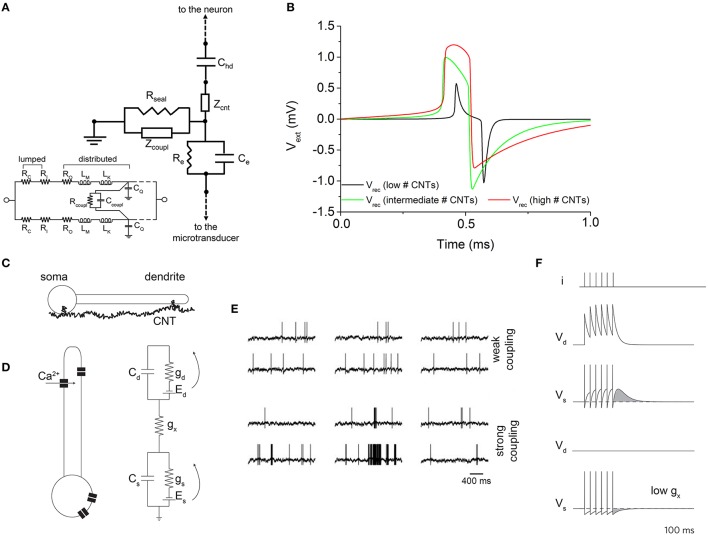
**Effect of CNTs on the recorded signal. (A)** Equivalent circuit which models the effect of vertically-aligned CNTs. Inset: equivalent circuit of two coupled CNTs. **(B)** Effect of the number of CNTs on the extracellular signal shape. An increase of the CNTs number (red line) makes the shape of the extracellular signal resembling the action potential. *Adapted from* Massobrio et al. (2008). **(C)** Cartoon describing the boosting activity induced by CNTs. **(D)** Bio-electrical models used to describe the genesis of the action potentials and the effect of CNTs. **(E)** Electrophysiological activity of the neuron model depicted in **(D)** under weak (top) and strong (bottom) coupling conditions. **(F)** Evaluation of the prolonged activity induced by the strong coupling (top), and comparison with the weak coupling (bottom). *Adapted from* Cellot et al. (2008).

Figure 7A shows the neuro-electronic junction circuit model of the coupling between a neuron and a CNT-functionalized microtransducer. This circuit configuration makes use of the main electrical components already introduced in the schematic of Figure 3. The inset of Figure 7A displays the RLC equivalent circuit model for two coupled CNTs, devised by Burke (2002, 2003). The impedance of an isolated CNT can be written as:

(6) $$Z_{CNT}=\frac{R_{CNT}+j\omega L_{CNT}}{{\left(j\omega \right)}^{2}\;L_{CNT}C_{CNT}+j\omega R_{CNT}C_{CNT}+1}$$

and the total coupling impedance of two neighboring CNTs can be evaluated as:

(7) $$Z_{COUPL}=\frac{R_{Coupl}-j\omega R_{Coupl}^{2}C_{Coupl}}{{\left(\omega R_{Coupl}C_{Coupl}\right)}^{2}+1}$$

Considering CNTs grown in vertical alignment and in normal direction to the microelectrode surface, they establish a bundle whose impedance (function of the number of the involved CNTs) can be split into two components: a CNTs bundle coupling impedance placed in parallel to the CNTs bundle top surface and which appears in parallel to *R*_*seal*_ in condition of weak coupling, or standalone in condition of strong coupling (*R*_*seal*_ → ∞), and a CNTs bundle impedance placed perpendicular to the CNTs bundle top surface (Massobrio et al., 2008, 2011). The dependence of these two impedances from the number of involved CNTs can be quantified as:

(8) $$Z_{COUPL}^{Bundle}=(n_{l}-1)\;\cdot \;(n_{w}-1)\;\cdot \;Z_{COUPL}$$

(9) $$Z_{CNT}^{Bundle}=\frac{Z_{CNT}}{n_{cnt}}.$$

where *n*_*w*_ and *n*_*l*_ are the number of “rows” and “columns” of CNTs in the bundle, and *n*_*cnt*_ is the total number of CNTs in the bundle. The number of CNTs coupled to the neuronal membrane seems to be the key parameter for understanding the recorded signal shape, as shown in Figure 7B. The proposed model predicts response amplitude larger than that recorded by the microtransducers not functionalized by CNTs (hundreds of microvolts). Moreover, for high values of *n*_*cnt*_, the expected recorded extracellular signal shape changes, resembling the intracellular membrane action potential (red curve in Figure 7B). The extracellular signal shape depends both on the electrical properties of the interface and on the electrical state of the membrane in the junction. The current flow over the seal resistor can be either capacitive or ohmic: in particular, when the capacitive current dominates, a biphasic shape is recorded; when a high ohmic current dominates, a monophasic shape is recorded. Jenkner and Fromherz (1997) reported that an increase in the conductance of the junction membrane facing the recording microtransducer surface, in concert with an increased seal resistance, leads to a transformation of the extracellular signal from a biphasic shape (proportional to the first derivative of the intracellular action potential), to a monophasic shape, which resembles in shape intracellularly recorded action potential: moreover, the signal amplitude is increased. Thus, a local increase in the junction membrane conductance associated with an increased *R*_*seal*_, transfers the capacitive coupling between the neuron and the recording microtransducer to ohmic coupling (Cohen et al., 2008). By enforcing these remarks to the considered CNTs-functionalized interface, a possible explanation of this configuration behavior can be attempted. In particular, under the conditions of a high number of CNTs in the bundle, the neuron would achieve a very tight coupling with CNTs, meaning that an ohmic coupling would prevail on the capacitive one. Thus, the shape of the recorded extracellular potential becomes similar to the intracellular one (Figure 7B).

Because of the tight contacts originated between CNTs and neuronal membrane, in 2005 Lovat and colleagues demonstrated CNTs were able to support dendrite elongation as well as to boost the electrophysiological activity by increasing the frequency of action potentials (Lovat et al., 2005). Some years later, a computational model was developed to explain this peculiar behavior induced by CNTs. In Cellot et al. (2008), the authors speculated the presence of electrical shortcuts between the compartments of the neuron adhered to a CNT-functionalized surface (Figure 7).

The authors developed a neuron model made up of two-compartments with two state-variables describing the voltage distributions of a somatic (*V*_*s*_) and a dendritic (*V*_*d*_) compartment (Figure 7D). *C*_*s*∕*d*_, *g*_*s*∕*d*_, as well as the voltage-sources *E*_*s*∕*d*_ are the somatic/dendritic membrane capacitances, conductances, and Nernst potentials, respectively tagged by the subscripts *s* and *d*. The inter-compartment conductance *g*_*x*_ takes into account the total charge transferred to the dendritic compartments by a back propagating action-potential and mediated by dendritic voltage-gated calcium channels (Figure 7E). Since *V*_*d*_ accumulates the contributions of closely fired somatic action potentials (Figure 7F), it acts as a kind of “charge reservoir,” which discharges back to the somatic compartment. When somato-dendritic coupling is weak, the dendritic compartment experiences almost no effect upon somatic spiking (Figure 7F, upper panel). On the contrary, when the dendrite is charged by somatic spikes, the model neuron fires spontaneously with the same rate, although some firing epoch might be prolonged by the extra depolarization (Figure 7F, lower panels).

### Gold engulfed mushroom-shaped microelectrode for recording synaptic signals

**Figure 8:**
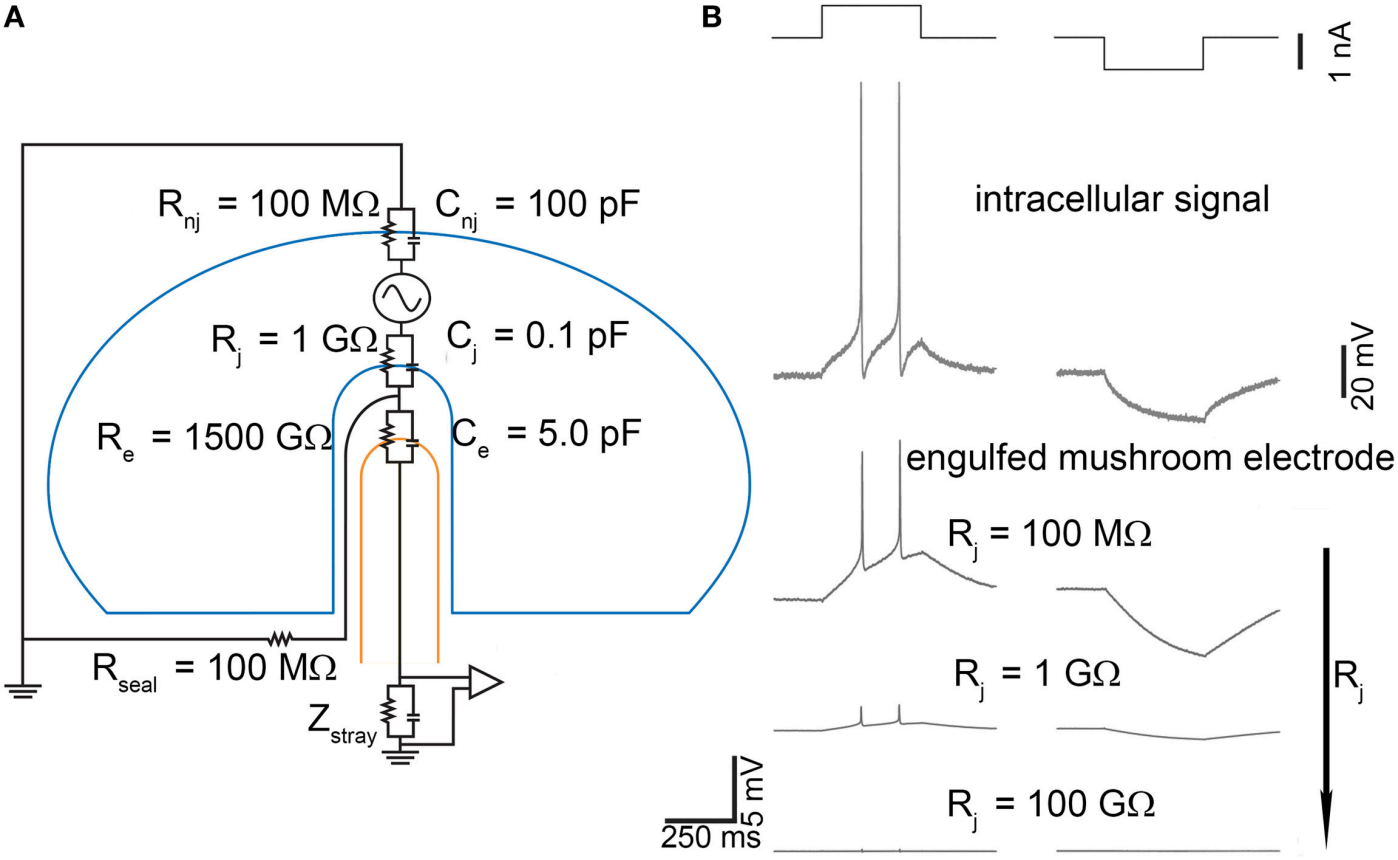
**Effect of gold-engulfed mushroom-shaped microelectrode on the recorded signal. (A)** Model equivalent circuit; **(B)** Simulations of two different electrophysiological signals (second row), action potentials, and hyperpolarization induced by positive and negative current pulses (first row). The amplitude of the signals recorded by the gold mushroom-shaped microelectrodes depends on the values of the junctional membrane resistance *R*_*x*_. *Adapted from* Spira and Hai (2013).

One of the main limitations of the extracellular microtransducers presented in the previous sections is their inability to record under-threshold signals, like post-synaptic potentials. Phenomena such as synaptic integration, disinhibition, under-threshold oscillations, cannot be recorded by these devices which only record strong supra-threshold signals like action potentials. To overcome such a limitation Spira's lab in 2007 (Spira et al., 2007) developed a new approach to improve and enhance the adhesion between the neuronal membrane and the sensing area of a microtransducer: a chemically functionalized micrometer-size mushroom-shaped gold protrusion as sensing electrode (Figure 1D) which may be engulfed by the cell. In this way, the coupling conditions are increased more than 400% (Spira and Hai, 2013). The first experimental attempts have been done by using large soma neurons extracted from invertebrates (*Aplysia californica*) and by coating with specific peptides the nano-electrode to favor the engulfment of the electrode into the membrane. The equivalent circuit model configuration (which describes this strong adhesion condition) is simple and made up of only passive electrical components (Figure 8A). Practically, the proposed model splits the neuronal membrane into two compartments: one coupled to the microtransducer (*R*_*j*_ and *C*_*j*_) and one uncoupled (*R*_*nj*_ and *C*_*nj*_). The other components of the circuit, namely the electrode-interface parallel *R*_*e*_–*C*_*e*_ and the sealing resistor *R*_*seal*_ maintain the same biophysical meaning described in the previous sections (cf., Section Equivalent Circuit of the Neuron-Microtransducer Interface).

As Figure 8A points out, the capacitive-resistive parallel modeling the neuron-microtransducer interface is also maintained, but it is “tailored” (in terms of sealing conditions) for this peculiar experimental configuration (i.e., gold engulfed mushroom). The key point of this device is the coupled membrane area which can range between very small portions of the cell surface area, up to large patches. These differences of coupling depend on the geometry of the microtransducer area and on the adhesion features of the neuronal membrane. Theoretically, the junctional membrane can present very high resistance and low capacitance. This implies that only a small fraction of the current generated across the neuron's membrane flows through the junctional membrane. Reduction of the junctional membrane resistance (*R*_*j*_ in the circuit model of Figure 8A) would be very effective in improving the electrical coupling between the neuron and the microelectrode. Several attempts have been made to enhance the coupling, such as manipulation of the ionic channels by means of an over-expression of ion channels in the neuronal membrane (Fromherz, 2007), or of localized electroporation (Xie et al., 2012). This condition has been recreated by using the gold engulfed mushroom-shaped microelectrodes functionalized by means of a peptide with multiple Arg-Gly-Asp repeats which facilitates the engulfment of the gold spines (Hai et al., 2009). Simulations of the model circuit of Figure 8A have been performed under different stimulation conditions (depolarization and hyperpolarization pulses, Figure 8B—top row) which elicit the action potentials and the voltage hyperpolarization depicted in the second row of Figure 8B. Lowering the junctional membrane resistance *R*_*j*_, the shape of the intracellular signal can be recorded (Figure 8B—bottom traces) with different attenuation values. Two major results can be found. First, the conservation of the intracellular signal shape: from the simulations, it comes out that the signal recorded by gold engulfed mushroom-shaped microelectrodes do not result in a kind of first (or second) derivative of the action potential as conventional planar microelectrodes do (Figures 4, 5). Second, also the hyperpolarized signal can be detected: although the amplitude of this passive potential is less than 20 mV, gold engulfed mushroom-shaped microelectrodes are able to reveal the presence of this kind of signal which cannot be recorded by any other kind of extracellular microtransducer.

The promising results obtained by using large invertebrate neurons (80 μm diameter) drove the same research-group to develop a second-generation of devices able to record the electrophysiological activity from small mammalian neurons (10–20 μm diameter) too (Ojovan et al., 2015). By maintaining the same electrical model circuit of Figure 8A, Ojovan et al. simulated the coupling of a hippocampal neuron with a peptide coated gold engulfed mushroom-shaped microelectrode, by taking into account the geometry and the size of the mushroom cap and stalk (Figure 1D). The authors considered two different experimental conditions: the ideal case of totally engulfed neurons, and the more realistic situation of partially engulfed neurons. Under these two different coupling conditions, they simulated mushroom-shaped microelectrodes by increasing: (i) the cap diameter (from 1.5 to 5 μm) and keeping constant (0.75 μm) the stalk diameter, or (ii) both the cap and the stalk diameters (maintaining the constraint that the cap diameter exceeds the stalk one by 1 μm). They found that the cap dimension plays a relevant role in the coupling: the larger the diameter, the higher is the value of the recorded signal. In contrast, the authors also found that an increase of the stalk diameter decreased the effect of increased cap diameter. These simulations, in conjunction with the morphological constraint of the cell dimensions of vertebrate neurons (10–20 μm diameter), suggest an upper limit of 2–2.5 μm for the cap diameter. However, such a value is not sufficient to record under-threshold potentials, which result too attenuate. The only possibility is to reduce the junctional membrane resistance. Simulations indicate a value of 50–80 MΩ to allow an “in-cell” recording (Ojovan et al., 2015) which can be achieved by the membrane curvature (Hai et al., 2010) or by the functionalization of the mushroom-shaped electrode (Khoutorsky et al., 2011).

## Discussions and conclusions

In this review, we presented some electronic circuit-based models of the neuro-electronic junction consisting of interfacing neurons to microtransducers in order to investigate the capability of such systems to record the neuronal electrophysiological activity. Despite their simplicity, the presented models, based on the concept of equivalent-circuits, well describe several experimental conditions recordings. The experimentally recorded shapes, though present a high degree of variability (duration, peak-to-peak amplitude, monophasic, or biphasic modes) are well fitted by the simulated signals only by changing the most significant coupling parameters and the ionic channel density at the neuron-to-microtransducer interface. Moreover, because of its simplicity, the proposed model allows to be suited to different types of microtransducer, ranging from metallic microelectrodes (Taketani and Baudry, 2006), to open-gate FETs (Fromherz et al., 1991), more complicated structures such as microtransducers functionalized by means of CNTs (Gabay et al., 2007; Galvan-Garcia et al., 2007; Massobrio et al., 2008), and gold engulfed mushroom-shaped devices (Hai et al., 2009). Such devices enable to characterize the neuronal dynamics of biological preparations,—ranging from invertebrates (Massobrio et al., 2009, 2013) to different cerebral mammalian areas [e.g., cortex (Pasquale et al., 2008), hippocampus (Brewer et al., 2009)] to study their development (Wagenaar et al., 2006)—to deliver electrical (Wagenaar et al., 2005) or chemical stimulations (Gross et al., 1995), to induce synaptic plasticity at the network level (Chiappalone et al., 2008). Compared to the first generation of FETs-based systems (Fromherz et al., 1991; Fromherz, 2003), nowadays low-noise level FETs-based systems are available: the introduction of low-noise transistors (Voelker and Fromherz, 2005) allow to record the electrophysiological activity also from mammalian neurons which exhibit peak-to-peak amplitude (~100 μV) smaller than that originated by large invertebrate neurons (up to tens of millivolts). In this way, the use of CNTs could be a valuable attempt to make FET-based devices able to record also small potential amplitudes. A lot of the research has been focused on CNTs as promising materials for the assembly of nano-devices: several laboratories are working on new CNT-composite materials in order to tailor CNT properties for specific applications. In particular, CNTs have been developed for medical and biotechnological applications including gene and drug delivery (Martin, 2006), enzyme immobilization and biosensing (Wang et al., 2005), microsurgery (Fortina et al., 2007), and electrophysiological neuronal activity investigation (Silva, 2006). In general, we can argue the application of CNTs to the nervous system is and it will be particularly suited to both basic neuroscience and clinical applications. On the other hand, gold engulfed mushroom-shaped microelectrodes (Hai et al., 2009) are an excellent approach for performing long-lasting recording, nevertheless maintaining the properties of the classical sharp intracellular microelectrodes or whole-cell patch clamp technique.

It is worth noticing that in the experimental configurations we discussed, the genesis of the extracellular signal is due to a capacitive contribution (i.e., displacement current) and to ionic currents where the channel density of the membrane coupled to the microelectrode is homogeneous with respect to the whole membrane. Indeed, a different scenario could appear if we hypothesize an increase-reduction of the channel density in the membrane patch (see for a detailed study, Buitenweg et al., 2002) or with the use of electroporation technique. A transient electroporation improves the quality (both shape and amplitude) of the recorded signal, thanks to a dramatically decrease of the coupling impedance. In this sense, the use of nano-pillar electrodes which can deliver large electric fields with a small voltage is another possible strategy to realize tight and not disruptive couplings (Xie et al., 2012).

As stated in the introduction, from a technical point of view, this review deals with only simple electrical circuit-equivalent models. The presented models and results have a first intrinsic limitation due to their non bi-directionality: the equivalent-circuits are able to describe the extracellular signals in the recording mode, but do not work in the stimulation mode. This operating condition requires considering different biophysical processes (and thus different electrical models), or different approaches have to be taken into account. Among these approaches, noteworthy are the one devised by McIntyre and co-workers based on *finite elements* models (Mcintyre and Grill, 2001, 2002) and the one based on the *linear volume conduction* theory formulated by Nunez and Srinivasan (2006). According to the latter approach, the extracellular potential is computed in two steps. First, the neuronal activity of a single neuron is simulated by means of *ad hoc* programs [e.g., Neuron (Carnevale and Hines, 2005)] and then the transmembrane currents are evaluated; second, the extracellular potential is computed as a weighted sum of all transmembrane currents in all the cells located close to the electrode. The main advantage of this approach lies in the possibility of taking into account the different frequency contributions (i.e., local field potentials and spiking activity; Einevoll et al., 2013; Lindén et al., 2014).

Finally, from a more general perspective, it is worth mentioning the increasing number of works dealing with the development of organic electronics-based devices that are becoming of great interest for neuro-electrophysiological applications (e.g., Khodagholy et al., 2013; Spanu et al., 2015). The theory used for modeling the coupling between such devices and neuronal membranes may still be applied for some kinds of organic devices, such as passive organic microelectrodes and organic FET-based devices, while for others (i.e., organic electrochemical transistors) a new specific modeling framework needs to be developed.

## Author contributions

PM, GM, and SM wrote the paper.

### Conflict of interest statement

The authors declare that the research was conducted in the absence of any commercial or financial relationships that could be construed as a potential conflict of interest.
